# Chemical stability of active ingredients in diluted veterinary disinfectant solutions under simulated storage conditions

**DOI:** 10.3389/fchem.2023.1204477

**Published:** 2023-06-16

**Authors:** Chae Hong Rhee, Hye-sook Lee, Hyeong-jun Yun, Ga-Hee Lee, Su-Jeong Kim, Sok Song, Myoung-Heon Lee, Moon Her, Wooseog Jeong

**Affiliations:** ^1^ Veterinary Drugs and Biologics Division, Animal and Plant Quarantine Agency, Gimcheon-si, Gyeongsangbuk-do, Republic of Korea; ^2^ Laboratory of Veterinary Pharmacokinetics and Pharmacodynamics, College of Veterinary Medicine, Kyungpook National University, Daegu, Republic of Korea; ^3^ Korea Animal Health Products Association, Seongnam-si, Gyeonggi-do, Republic of Korea

**Keywords:** content analysis, disinfectant, chemical stability, HPLC, active ingredient, titration, icp-oes

## Abstract

**Introduction:** The product labels of veterinary disinfectants specify their expiration dates to prevent the use of outdated products, as these may result in disinfection and biosecurity failures during outbreak situations. However, a clear standard for the storage conditions of diluted disinfectant solutions has not yet been established, and the effects of storage conditions have scarcely been investigated. To fill this research gap, our study examined the stability of the active ingredients of diluted veterinary disinfectants based on their change in concentrations when stored at various temperatures for various time periods.

**Methods:** Twenty veterinary disinfectants effective against either foot-and-mouth disease or avian influenza viruses were selected. The disinfectants were diluted to effective concentrations following the manufacturer’s instructions. Using selective analytical techniques, the concentrations of the active ingredients of the samples that had been stored for varying intervals at different temperatures (4, 20, 30, and 45°C) were determined. These samples included soaps and detergents, acids, oxidizing agents, aldehydes, and copper compounds. The active ingredient concentrations of two of the samples were determined following freezing/thawing cycle, to establish their stability when exposed to simulated winter conditions.

**Results:** Our results showed that most of the active ingredients had concentrations of 90% or greater of their initial concentrations, indicating ≥90% stability over a 21-day period under the experimental storage conditions. However, there were some exceptions. Glutaraldehyde, formaldehyde, and malic acid are over 90% stable at ≤ 30°C for 21 days, but their concentrations decreased to below 90% of their initial concentrations at 45°C, indicating a decline in stability when stored at 45°C for 21 days. The concentrations of potassium peroxymonosulfate and peracetic acid rapidly declined with increasing time and temperature to less than 90% of their initial concentrations.

**Discussion:** Based on our findings, we propose that diluted disinfectant solutions should preferably be prepared daily. However, if the daily preparation of a diluted disinfectant solution is not feasible, then our results can be used as a reference, providing basic scientific data on the chemical stability of diluted disinfectant solutions commonly used in the veterinary field, thus indicating suitable storage conditions.

## 1 Introduction

The active ingredients in a disinfect are the chemicals responsible for inhibiting the growth of microorganisms. The contents of these ingredients are listed as percentages or concentrations on the product label, along with their respective expiration dates. The expiry period is based on the stability of the active ingredients, known to decline over time owing to several chemical processes triggered by various conditions.

In South Korea, the Animal and Plant Quarantine Agency (APQA) performs quality control of commercial veterinary disinfectants, both through an official approval step and an annual post-market surveillance study, evaluating their efficacy against the targeted microorganisms and the contents of the active ingredients in the final product (Rhee et al., 2022). Currently, the efficacy against the targeted microorganism is assessed by the dilution concentration indicated on the label; however, the content of the active ingredient in the final product is used to determine whether it conforms to the stability specification of 90%–120% of the label claim (Rhee et al., 2022).

Disinfectants are classically grouped into soaps and detergents, alkalis, acids, chlorine and chlorine compounds, oxidizing agents, aldehydes, phenol compounds, quaternary ammonium compounds (QACs), and alcohols (Maris, 1995; Geering et al., 2001; De Benedictis et al., 2007). The World Organization for Animal Health (OIE) has grouped viruses into three categories, denoted as A, B, and C, in terms of resistance to chemical agents, and has presented the best type of disinfectant to use on a range of commonly contaminated items for each disease or group of diseases (Geering et al., 2001). The choice of disinfectant for a specific application is critical in achieving maximum effectiveness. In addition, an appropriate concentration of a disinfectant is important to achieve optimal results in an outbreak situation, whereas the stability of its active ingredient is crucial to ensure the efficacy of the disinfectant throughout its shelf life (Farrag et al., 2023). Stability of the active ingredient is one of many factors that influence the overall stability of a disinfectant product (Singh and Bakshi, 2000; Bajaj et al., 2012; Farrag et al., 2023).

Numerous studies have investigated the efficacy of active ingredients or disinfectants against microorganisms; at specific concentrations, for given time intervals and/or various temperatures (Muhammad et al., 2001; Yilmaz et al., 2004; De Benedictis et al., 2007; Jang et al., 2014; Singh et al., 2017; Lin et al., 2020; Pironti et al., 2021; Kobayashi et al., 2022; Xiao et al., 2022). Phenol compounds have been tested for their ability to inactivate the highly pathogenic avian influenza virus (HPAIV) at different concentrations (0.1%, 0.2% and 0.4%) and for different contact times (6, 12, 18 and 24 h) (Muhammad et al., 2001). The efficacies of chlorhexidine gluconate and ethanol, the active ingredients of hand sanitizers, have been investigated by a time-kill test against bacteria and fungi (Kobayashi et al., 2022), whereas the efficacy of a commercial organic acid containing disinfectant against a low pathogenic avian influenza virus (LPAIV) has been investigated at different concentrations (0.1%, 0.5%, 1% and 2%) and temperatures (4, 10 and 20°C), and for different contact times (5, 10, 15, 30, 60 and 120 min) (Yilmaz et al., 2004). Singh et al. (2017) have evaluated the virucidal efficacy of commercial disinfectants belonging to chlorine, phenol, and a combination of QACs and aldehydes, against the Senecavirus A at 4°C–25°C with contact intervals of 1–15 min. Jang et al. (2014) have evaluated the changes in the efficacies of disinfectants belonging to acids, oxidizing agents, and aldehydes at different temperatures (25, 4, 0 and −10°C) and different contact times (1 and 5 min) to establish the optimal conditions for disinfection against H9N2 LPAIV. Pironti et al. (2021) have assessed the antimicrobial properties of permaleic acid (PMA) and peracetic acid (PAA) against *Escherichia coli* and *Staphylococcus aureus* at concentrations ranging from 10 to 0.002 g/L and at contact times of 30, 60 and 90 min. Other authors have summarized the studies on the common chemical disinfectants and their virucidal efficacies under various application conditions. Lin et al. (2020) have reviewed the effectiveness of disinfectants against viruses under various conditions, such as concentration, exposure time duration, and temperature, whereas Xiao et al. (2022) have reviewed the virucidal efficacy of disinfectants against SARS-CoV-2. Xiao et al. have included the types and concentrations of the chemical disinfectants, the formulations, and the exposure times in their review.

However, research on the stability of active ingredients, based on concentration changes in diluted veterinary disinfectant solutions under certain storage conditions, is scarce and there is a lack of scientific evidence regarding the relationship between the actual concentration of active ingredients in diluted disinfectant solutions and their efficacies. Moreover, as no clear standard has yet been established for the storage conditions of diluted disinfectant solutions, daily replacement and preparation of fresh dilutions are recommended in most disinfection protocols to ensure optimal efficacy (Dvorak, 2008; Rutala and Weber, 2008). In infectious animal disease outbreaks, disinfectants are used in large quantities outdoors. The required amounts depend on various factors, such as the target organisms, degree of traffic flow, area size, and organic matter. In such situations, the daily preparation of a diluted disinfectant solution may not be feasible.

In this study we attempted to elucidate how the concentrations of the active ingredients of diluted disinfectant solutions and therefore their stabilities change under various storage conditions. The study included the assessment of the stability of diluted disinfectant solutions when exposed to a freezing/thawing cycle, to investigate the stability of diluted disinfectant solutions during January, known as the coldest month in South Korea with average temperatures reaching −6.9°C to 3.6°C (KMA, 2023). In the winter season, heat wires, thermostats, indoor storage, and other means are used to prevent freezing of the diluted disinfectant solutions as their stability when frozen has not been confirmed scientifically. We evaluated diluted disinfectant solutions of various chemical compositions in our study, including soaps and detergents, acids, oxidizing agents, aldehydes, and copper compounds, under various storage conditions (Table 1). These conditions were chosen to assess how specific storage conditions such as temperatures and storage times affect the efficacy of disinfectants. Concentration-based assays were performed to determine the changes in the contents of the active ingredients, thereby generating basic scientific data to establish the stabilities of the active ingredients, and to suggest suitable storage conditions for the diluted disinfectant solutions.

**TABLE 1:** Description and contents of active ingredients in the twenty disinfectants used in this study.

Classification	Disinfectant	Active ingredient		Content on label^*^	Active concentration^**^ (%)	Analytical application
Soaps and detergents	A	Quaternary ammonium chloride	Didecyl dimethyl ammonium chloride	5% (w/v)	0.100	Titration
Combinations (Soaps and detergents + Acids)	B	Quaternary ammonium chloride	Alkyl dimethyl benzyl ammonium chloride	10% (w/v)	0.063	Titration
		Citric acid		20% (w/v)	0.125	HPLC
Combinations (Soaps and detergents + Aldehydes)	C	Quaternary ammonium chloride	Alkyl dimethyl benzyl ammonium chloride	6% (w/v)	0.040	Titration
		Glutaraldehyde		5% (w/v)	0.033	HPLC
		Formaldehyde		8% (w/v)	0.053	HPLC
Combinations (Soaps and detergents + Aldehydes)	D	Quaternary ammonium chloride	Alkyl dimethyl benzyl ammonium chloride	10% (w/v)	0.042	Titration
		Citric acid		30% (w/v)	0.125	HPLC
Combinations (Soaps and detergents + Acids)	E	Quaternary ammonium chloride	Alkyl dimethyl benzyl ammonium chloride	10% (w/v)	0.063	Titration
		Citric acid		20% (w/v)	0.125	HPLC
		Phosphoric acid		10% (w/v)	0.063	ICP-OES
Combinations (Soaps and detergents + Aldehydes)	F	Quaternary ammonium chloride	Coco dimethyl benzyl ammonium chloride	10% (w/v)	0.156	Titration
		Glutaraldehyde		15% (w/v)	0.234	Titration
Acids	G	Citric acid		50% (w/v)	0.333	HPLC
Acids	H	Citric acid		50% (w/v)	0.313	HPLC
Acids	I	Citric acid		40% (w/v)	0.267	HPLC
Acids	J	Citric acid		20% (w/v)	0.080	HPLC
		Acetic acid		10% (w/v)	0.040	HPLC
		Phosphoric acid		10% (w/v)	0.040	ICP-OES
Oxidizing agents	K	Potassium peroxymonosulfate		50% (w/w)	0.250	Titration
Oxidizing agents	L	Potassium peroxymonosulfate		50% (w/w)	0.250	Titration
Oxidizing agents	M	Potassium peroxymonosulfate		50% (w/w)	0.250	Titration
		Sodium dichloroisocyanurate		5% (w/w)	0.025	
Oxidizing agents	N	Potassium peroxymonosulfate		50% (w/w)	0.500	Titration
		Malic acid		10% (w/w)	0.100	HPLC
Oxidizing agents	O	Hydrogen peroxide		25% (w/w)	0.031	Titration
		Peracetic acid		5% (w/w)	0.006	Chronoamperometry
Oxidizing agents	P	Hydrogen peroxide		27.5% (w/v)	0.034	Titration
		Peracetic acid		5.8% (w/v)	0.007	Chronoamperometry
Oxidizing agents	Q	Hydrogen peroxide		27.5% (w/v)	0.046	Titration
		Peracetic acid		5.8% (w/v)	0.010	Chronoamperometry
Aldehydes	R	Glutaraldehyde		10% (w/v)	0.200	Titration
Oxidizing agents	S	Sodium hypochlorite (4%–6%)		99% (w/v)	0.079–0.119	Titration
Copper compound	T	Copper sulfate pentahydrate		20% (w/w)	0.318	Titration

* Concentration of active ingredient.

** Concentration of active ingredient in the diluted disinfectant solution used in the experiments.

## 2 Materials and methods

### 2.1 Disinfectants

Twenty veterinary disinfectants, approved by the APQA as effective against either foot-and-mouth disease virus (FMDV) or avian influenza virus (AIV), were selected and classified according to their major active ingredients. The categories of active ingredients in disinfectants are detergents, acids, oxidizing agents, aldehydes, and copper compounds. A summary of the disinfectants and the classification of chemical disinfectants is presented in Table 1. All disinfectants were diluted with tap water to obtain the authorized effective concentrations against the targeted pathogens, and defined as diluted disinfectant solutions.

### 2.2 Stability testing conditions

The stability of the active ingredients in the 20 diluted disinfectant solutions was studied at four temperatures (4, 20, 30 and 45°C) and at different time intervals (0, 1, 2, 3, 5, 7, 10, 14 and 21 days). The active ingredient of each sample was assessed immediately after preparation (Day 0), prior to storage at the set temperature, and again after removal from storage at the set time intervals, using selective analytical techniques. Additionally, two diluted disinfectant solutions were assayed for changes in the content of active ingredients during a freezing/thawing cycle, i.e., the solutions were frozen after testing on the day of dilution (Day 0), thawed on the next day (Day 1), tested, and frozen again. The active ingredient of a sample was considered stable if it was within 90%–120% of its initial concentration.

### 2.3 Content analysis applications

#### 2.3.1 Chemical reagents

U.S. Pharmacopeia (USP) reference standards for citric acid (CA, ≥99% purity), malic acid (MA, ≥99% purity), acetic acid (AA, ≥99% purity), glutaraldehyde (GLT, Grade I, 25% in H_2_O), and formaldehyde (FAL, 37% stabilized with methanol) were used for high-performance liquid chromatography (HPLC) analysis. A phosphorus standard solution (1,000 mg/L) was purchased from Sigma–Aldrich (St. Louis, MO, United States) for inductively coupled plasma optical emission spectroscopy (ICP-OES). HPLC-grade water was obtained from Honeywell Burdick and Jackson (Morristown, NJ, United States). For the titration method validation, potassium peroxymonosulfate (PPMS, 99% purity), hydrogen peroxide (H_2_O_2_, 35% in H_2_O), and GLT (Grade I, 25% in H_2_O) were purchased from Sigma–Aldrich, and other active ingredients were obtained from the manufacturers. The standard solutions of quaternary ammonium compounds (QACs), sodium hypochlorite (NaOCl, 5.50% available chlorine), and copper sulfate pentahydrate (CuSO4.5H_2_O, 99.6% purity) were provided by the manufacturers. The QACs of didecyl dimethyl ammonium chloride (DDAC), alkyl dimethyl benzyl ammonium chloride (ADBAC), and coco dimethyl benzyl ammonium chloride (CDBAC) were of 81%, 50.58%, and 50.12% purity, respectively. All other reagents were of analytical grade.

#### 2.3.2 Titration

Determination of the QACs, GLT, PPMS, sodium dichloroisocyanurate (NaDCC), H_2_O_2_, NaOCl, and CuSO_4_.5H_2_O were directly performed via titration using an automated titrator (888 Titrando, Metrohm, Herisau, Switzerland), according to the previously validated and/or slightly modified APQA procedures (APQA, 2015; APQA, 2017; Rhee et al., 2022). Supplementary Figures S1, S2 illustrate the analytical procedure for the titration methods. The QACs were quantified using three different manual titration procedures: DDAC was determined using a titrant of silver nitrate (Supplementary Figure S1); ADBAC was determined using a titrant of sodium tetraphenylborate solution (Supplementary Figure S1); and CDBAC was determined using a titrant of sodium lauryl sulfate (Supplementary Figure S1). The concentrations of GLT and H_2_O_2_, as the single active ingredient in the disinfectant, were determined using a titration method (Supplementary Figure S2). Available oxygen was determined for the content analysis of PPMS and/or a combination of PPMS and NaDCC (Supplementary Figure S2), and available chloride was determined for NaOCl content analysis (Supplementary Figure S2). The concentration of copper sulfate (CuSO_4_) was determined for CuSO_4_.5H_2_O content analysis (Supplementary Figure S2). Each experiment was performed in triplicate, and the average values were used for analysis. The results were expressed as a percentage of the results obtained on Day 0 (baseline).

#### 2.3.3 HPLC-DAD analysis

All samples taken from the diluted disinfectant solutions were filtered through a 0.45 µm membrane filter (Millipore, Bedford, MA, United States) before injection. An HPLC system (Agilent 1,260 Infinity II series, United States) equipped with a pump (Model G7111 A), autosampler (Model G7129 A), sample thermostat (Model G4761 A), column oven (Model G7116 A), and a diode array detector (DAD; Model G7117C) was used to analyze CA, MA, AA, GLT, and FAL. Chemstation Software (Version Rev C.01.10 (236)) was used for data processing and evaluation. The analytical chromatographic separations of CA, MA, and AA were performed on a Zorbax SB-Aq column (4.6 × 150 mm, 5 µm), with the following conditions. A mobile phase composed of a 50 mM potassium phosphate buffer (KH_2_PO_4_) adjusted to pH 2.0 with phosphoric acid; flow rate of 1.0 mL/min; column compartment temperature of 35°C; injection volume of 10 μL; and ultraviolet (UV) detection at 210 nm. The analytical chromatographic separations of GLT and FAL were performed after derivatization with 2,4-dinitrophenylhydrazine (DNPH), on a Zorbax SB-Aq column (4.6 × 150 mm, 5 µm) with a mobile phase composed of acetonitrile and water (72:28 v/v); flow rate of 1.0 mL/min; column compartment temperature of 40°C; injection volume of 5 μL; and UV detection at 360 nm. The concentrations of the active ingredients were obtained using a calibration curve. Each experiment was performed in triplicate, and the average values were used for analysis. The results were expressed as a percentage of the results obtained on Day 0 (baseline).

#### 2.3.4 ICP-OES analysis

The phosphoric acid (PA) content was determined by quantifying the phosphorus in the samples, using an ICP-OES (Perkin Elmer, United States, Model Optima 2100 DV). Phosphorus was determined in the axial configuration, and argon (99.99 v/v%) was used for plasma generation at a flow rate of 1.0 mL/min. The spectral wavelength selected for phosphorus analysis was 213.617 nm. The phosphorus concentration was obtained using a calibration curve. Each experiment was performed in triplicate, and the average values were used for analysis. The results were expressed as a percentage of the results obtained on Day 0 (baseline).

#### 2.3.5 Chronoamperometry measurement

To measure the concentration of peracetic acid (PAA), the chronoamperometry test Kemio™ (Palintest, Gateshead, United Kingdom) with corresponding sensors for PAA was used. Each experiment was performed in triplicate, and the average values were used for analysis. The results were expressed as a percentage of the results obtained on Day 0 (baseline).

### 2.4 Data analysis

Data analyses were performed using Microsoft Excel 2019. All data were expressed as the mean, and significant differences were calculated using an ANOVA. Linear regression analyses were performed on Excel, and the 95% confidence intervals (CIs) were calculated using a reported formula (Hossain et al., 2013). The active ingredient contents obtained on each day of analysis were converted to a percentage of that measured on Day 0 (baseline). For each active ingredient, the time dependence of the mean percentage of the Day 0 concentration was determined, and the active ingredient was considered stable if the sample was within 90%–120% of its initial concentration. Content stability was reported as the number of days in which the lower level of the 95% CI for each ingredient at each temperature remained at or above 90% of the initial baseline concentration, indicating ≥90% stability.

## 3 Results

A total of 14 active ingredients in 20 diluted disinfectant solutions were analyzed for content stability in this study: QACs including DDAC, ADBAC, and CDBAC; acids including CA, MA, PA, AA, and PAA; oxidizing agents including PPMS, H_2_O_2,_ and NaOCl; aldehydes including GLT and FAL; and CuSO_4_.5H_2_O.

### 3.1 Analytical method validation

The analytical methods were validated to demonstrate their suitability for the intended purposes.

#### 3.1.1 Titration methods

For the titration method, the titrant volume at the end-point increased linearly with increasing concentrations for each active ingredient. Five tests were conducted for each titration assay, over a standard concentration range covering the concentrations of the compounds in the samples; the regression coefficients (*R* ^2^) were ≥0.9998 in all cases. The standard concentration ranges were 10–100 mg for DDAC, 1–10 mg for ADBAC, 5–50 mg for CDBAC, 10–80 mg for GLT, 2–30 mg for PPMS, 5–75 mg for H_2_O_2_, 5.5–60 mg for NaOCl, and 5–40 mg for CuSO_4_.5H_2_O. Precision was expressed as a percentage relative standard deviation (RSD%), and the obtained RSD% values were: ≤ 1% for DDAC, ≤ 0.5% for ADBAC, ≤ 1.56% for CDBAC, ≤ 0.5% for GLT, < 1% for PPMS, ≤ 1.5% for H_2_O_2_, ≤ 1.2% for NaOCl, and ≤ 0.8% for CuSO_4_.5H_2_O. These results represent both intra-day and inter-day performances, indicating an acceptable degree of precision for all the titration methods.

#### 3.1.2 HPLC methodology

Table 2 presents the analytical parameters, including linearity, limit of detection (LOD), limit of quantification (LOQ), precision, and accuracy, obtained for the validation of the HPLC method used for AA, CA, MA, GLT, and FAL determinations. Calibration graphs were prepared over a 5-point standard concentration range. All compounds exhibited linear relationships between concentrations and instrument responses, with regression coefficients of ≥0.9999, and the methods showed adequate precision. The RSD values were within 0.90% and 0.58%, the mean recoveries of the standard solutions were within 99.28%–103.40% and 98.04%–101.40%, and the LODs ranged from 0.32 to 0.55 μg/mL and 0.14 to 0.17 μg/mL, for the acids and aldehydes, respectively.

**TABLE 2:** Analytical features of the method used for the determination of acids and aldehydes using HPLC.

Active ingredient	Linearity			LOD (µg/mL)	LOQ (µg/mL)	Accuracy			Precision		
	Concentration (µg/mL)	Regression equation	*R* ^2^*			Added concentration (µg/mL)	Recovery mean (%) ± SD	RSD (%)	Amount (g)	Measured mean (%) ± SD	RSD (%)
Acetic acid	2–20	y = 0.6785x + 0.1136	0.9999	0.55	1.68	30	101.58 ± 0.42	0.35	1.0	97.73 ± 0.22	0.22
(AA)						50	102.76 ± 0.48	0.34	2.0	96.99 ± 0.23	0.23
						100	100.19 ± 0.35	0.19	3.0	97.08 ± 0.20	0.21
Citric acid	2–20	y = 1.2856x + 0.198	1.0000	0.54	1.65	30	103.40 ± 0.71	0.44	0.1	101.69 ± 0.81	0.81
(CA)						50	99.28 ± 1.28	0.72	0.2	101.13 ± 0.90	0.90
						100	99.75 ± 1.60	0.70	0.3	101.45 ± 0.26	0.26
Malic acid	2–20	y = 1.046x + 0.101	1.0000	0.32	0.97	30	98.10 ± 0.09	0.07	10.0	100.63 ± 0.27	0.27
(MA)						50	100.74 ± 0.39	0.26	20.0	99.41 ± 0.32	0.32
						100	100.69 ± 0.17	0.08	30.0	98.40 ± 0.20	0.20
Glutaraldehyde	1–10	y = 183.55x—9.4153	1.0000	0.17	0.51	1.05	100.00 ± 0.0131	0.43	1.0	100.00 ± 0.1660	0.17
(GLT)						2.11	101.40 ± 0.0134	0.33	1.5	101.36 ± 0.5828	0.58
						4.22	102.80 ± 0.0121	0.19	2.0	100.27 ± 0.0958	0.10
Formaldehyde	1–10	y = 342.30x + 14.264	1.0000	0.14	0.42	1.02	98.04 ± 0.0051	0.13	1.0	100.00 ± 0.1080	0.11
(FAL)						2.04	99.02 ± 0.0054	0.11	1.5	101.46 ± 0.1080	0.11
						4.09	100.49 ± 0.0033	0.05	2.0	100.18 ± 0.0624	0.06

Abbreviations: LOD, limit of detection; LOQ, limit of quantification; SD, standard deviation; RSD, relative standard deviation.

^a^Regression coefficient.

#### 3.1.3 ICP-OES methodology

The ICP-OES method, employed for determining the phosphorus content in the PA of the samples, was validated for linearity, LOD, LOQ, precision, and accuracy using standard PA solutions. A linear relationship was obtained over the five-point standard concentration range (0.1–10 μg/mL) with a regression equation of y = 38534x + 387.7 and, a regression coefficient of 0.9998. Intra-assay repeatability was assessed in one laboratory by a single operator, by testing identical samples (*n* = 10) of 0.02 μg/mL; the RSD% was 4.72%. The mean recoveries (±SD) and RSD% of the standard solutions were: 94.80% ± 10.31% and 10.87% for 0.1 μg/mL, 106.70% ± 3.56% and 3.33% for 1 μg/mL, and 104.29% ± 0.71% and 0.68% for 5 μg/mL. The LOD and LOQ of the phosphorus content were 0.0037 and 0.0110 μg/mL, respectively.

### 3.2 Contents of active ingredients

#### 3.2.1 Stable active ingredients

##### 3.2.1.1 QAC content

**FIGURE 1:**
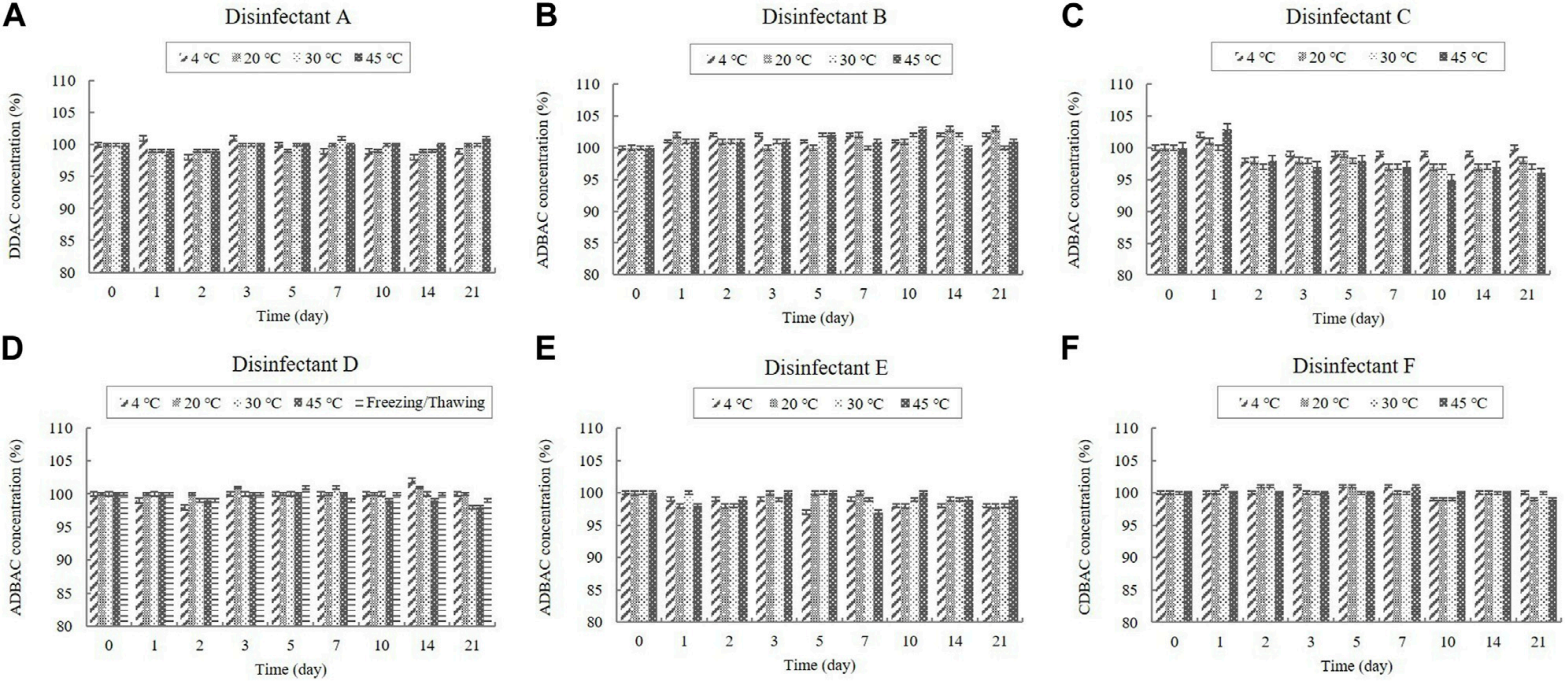
Stability of quaternary ammonium compounds (QACs) at various temperatures over time. QAC concentration in the diluted **(A)** disinfectant A solution, **(B)** disinfectant B solution, **(C)** disinfectant C solution, **(D)** disinfectant D solution, **(E)** disinfectant E solution, and **(F)** disinfectant F solution. Concentration is expressed as a percentage of the concentration measured at study initiation. Here, the abbreviations represent: DDAC, didecyl dimethyl ammonium chloride; ADBAC, alkyl dimethyl benzyl ammonium chloride; CDBAC, coco dimethyl benzyl ammonium chloride.

Disinfectants A, B, C, D, E, and F are QAC-based disinfectants that either contain a single active ingredient or a combination of acids and aldehydes. QACs are present in diluted disinfectant solutions at concentrations of 0.03%–0.16% (Table 1). The QAC concentrations in the samples of the diluted disinfectant D solution exhibited no significant change at any temperature during the experimental storage period, nor when subjected to a freezing/thawing cycle (Figure 1).

##### 3.2.1.2 CA, AA, and PA contents

**FIGURE 2:**
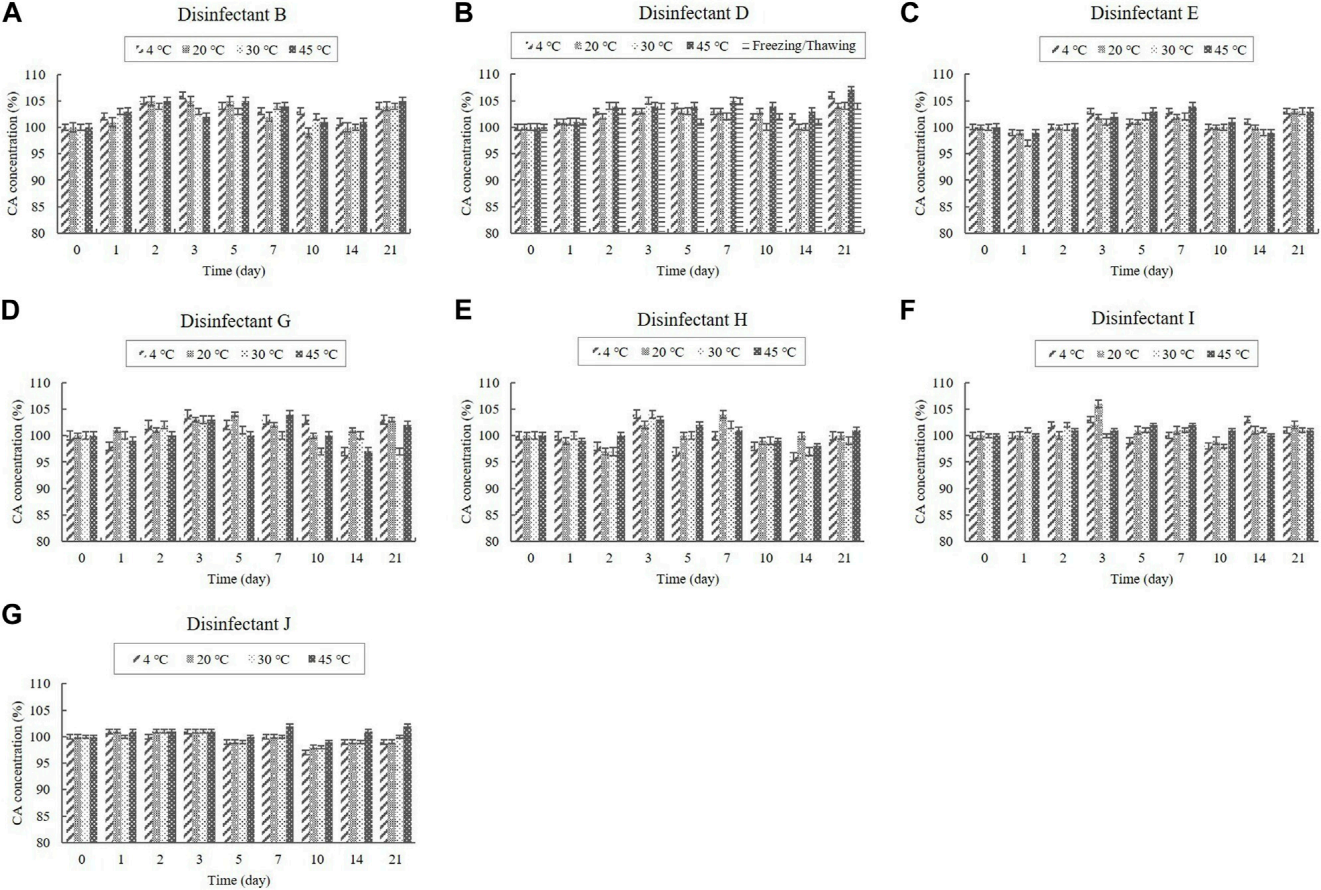
Stability of citric acid (CA) at various temperatures over time. CA concentration in the diluted **(A)** disinfectant B solution, **(B)** disinfectant D solution, **(C)** disinfectant E solution, **(D)** disinfectant G solution, **(E)** disinfectant H solution, **(F)** disinfectant I solution, and **(G)** disinfectant J solution. Concentration is expressed as a percentage of the concentration measured at study initiation.

**FIGURE 3:**
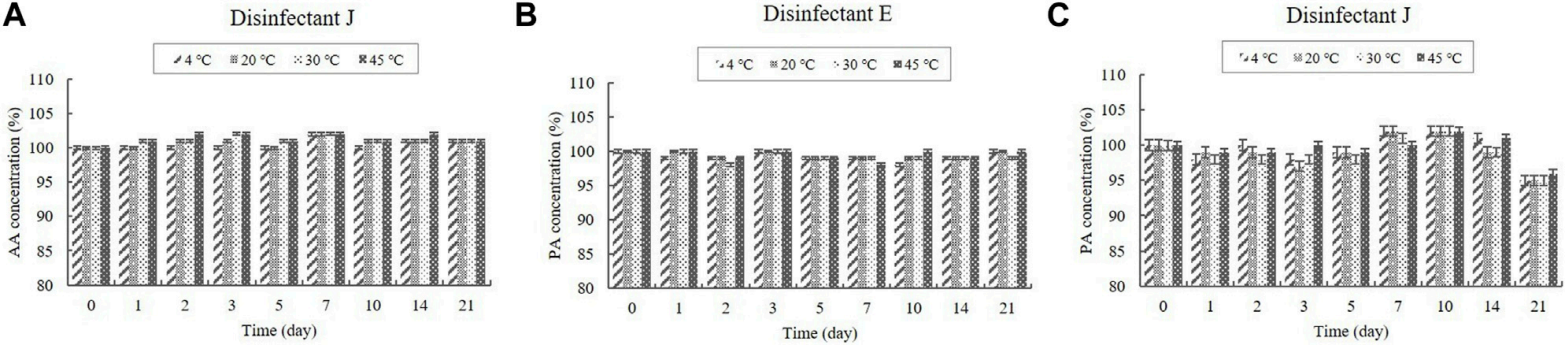
Stability of acetic acid (AA) and phosphoric acid (PA) at various temperatures over time. **(A)** AA concentration in the diluted disinfectant J solution. PA concentration in the diluted **(B)** disinfectant E solution and **(C)** disinfectant J solution. Concentration is expressed as a percentage of the concentration measured at study initiation.

The contents of the organic acids (CA and AA) were determined using HPLC, whereas the content of the inorganic acid (PA) was determined using ICP-OES. CA was present in the diluted disinfectant solutions of disinfectants B, D, E, G, H, I, and J at concentrations of 0.08%–0.33%. The diluted disinfectant solution of disinfectant J also contained AA at a concentration of 0.040% (Table 1). PA was present in the diluted disinfectant solutions of disinfectants E and J at concentrations of 0.06% and 0.04%, respectively (Table 1). The CA, AA, and PA concentrations in the samples did not change significantly at any of the temperatures during the experimental storage period. Moreover, the CA concentration in the samples of the diluted disinfectant D solution did not change significantly when exposed to a freezing/thawing cycle (Figure 2; Figure 3).

##### 3.2.1.3 H_2_O_2_ content

**FIGURE 4:**
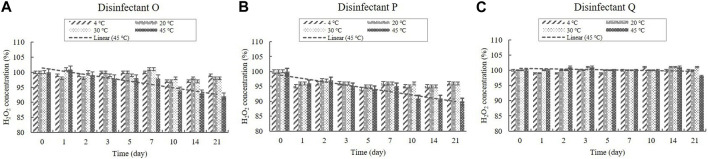
Stability of H_2_O_2_ at various temperatures over time. H_2_O_2_ concentration in the diluted **(A)** disinfectant O solution, **(B)** disinfectant *p* solution, and **(C)** disinfectant Q solution. Concentration is expressed as a percentage of the concentration measured at study initiation. A linear trend line fit (dotted line) of the 45°C data is displayed.

H_2_O_2_ was present in the diluted disinfectant solutions of disinfectants O, P, and Q at concentrations of 0.03%–0.05% (Table 1). The H_2_O_2_ concentration in the samples did not change significantly at 4, 20, and 30°C during the experimental storage period (Figure 4). However, the concentrations decreased significantly (*p* < 0.05) at 45°C from Day 10 onwards in the samples of disinfectants O and P. Nevertheless, the concentrations were above 90% of the initial concentrations at the end of the experimental storage period (Figure 4).

##### 3.2.1.4 NaOCl and CuSO_4_ contents

**FIGURE 5:**
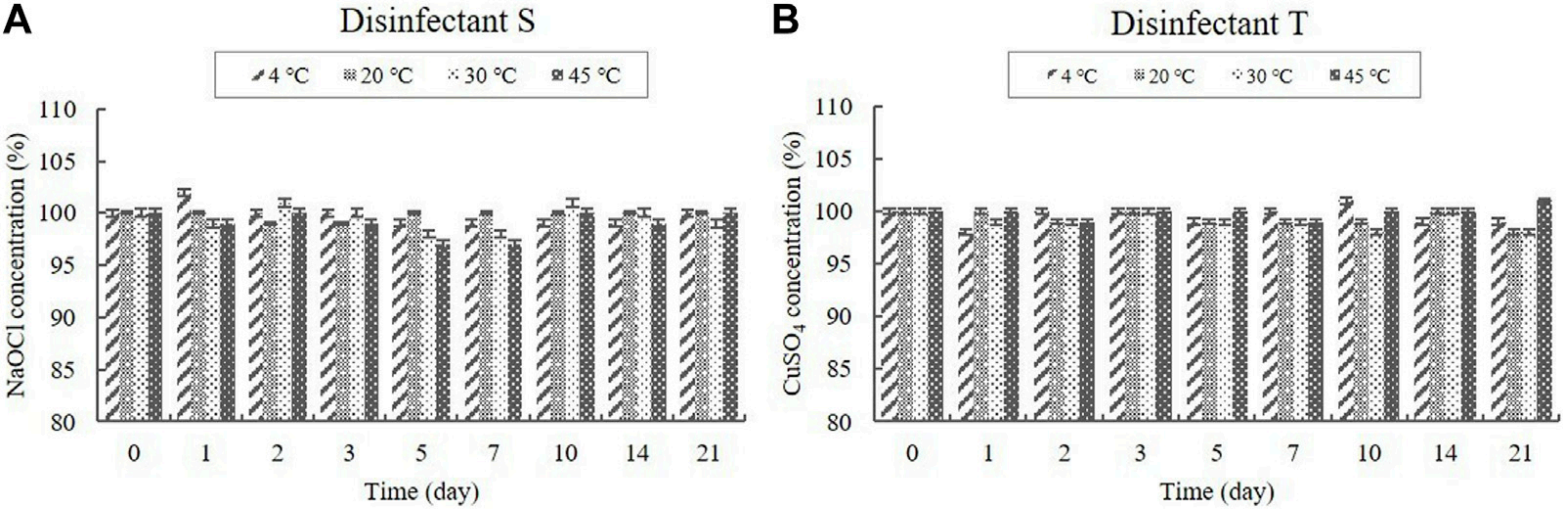
Stability of NaOCl and CuSO_4_ at various temperatures over time. **(A)** NaOCl concentration in the diluted disinfectant S solution, **(B)** CuSO_4_ concentration in the diluted disinfectant T solution. Concentration is expressed as a percentage of the concentration measured at study initiation.

NaOCl was present in the diluted disinfectant solution of disinfectant S at a concentration of 0.1%, whereas CuSO_4_ was present in the diluted disinfectant solution of disinfectant T at a concentration of 0.318% (Table 1). The concentrations in the samples did not change significantly at any of the temperatures during the experimental storage period (Figure 5).

#### 3.2.2 Content changes of active ingredients on conditional change

##### 3.2.2.1 GLT and FAL contents

**FIGURE 6:**
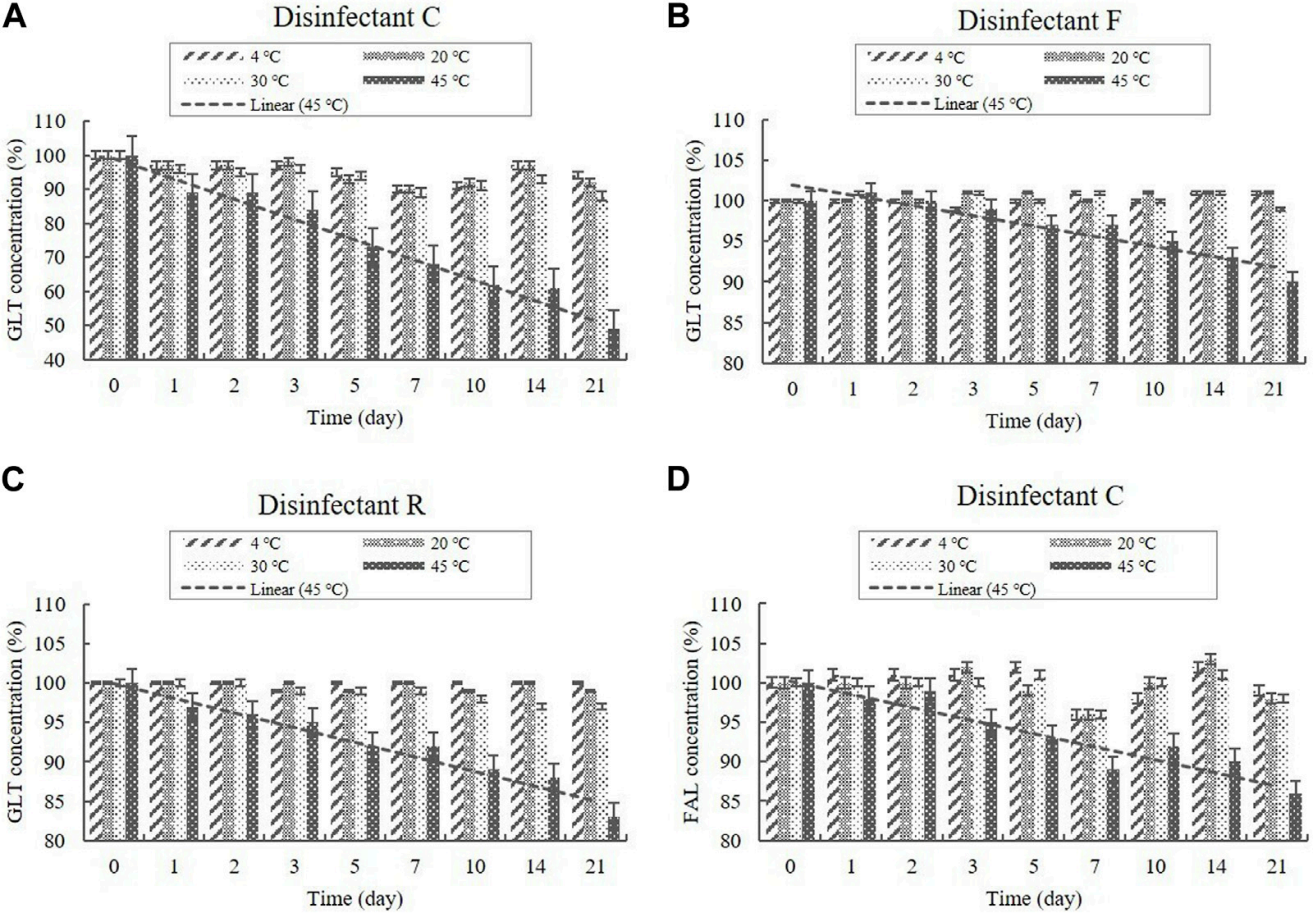
Stability of GLT and FAL at various temperatures over time. GLT concentration in the diluted **(A)** disinfectant C solution, **(B)** disinfectant F solution, and **(C)** disinfectant R solution. **(D)** FAL concentration in the diluted disinfectant C solution. Concentration is expressed as a percentage of the concentration measured at study initiation. Linear trend line fit (dotted line) of the 45°C data is displayed.

**FIGURE 7:**
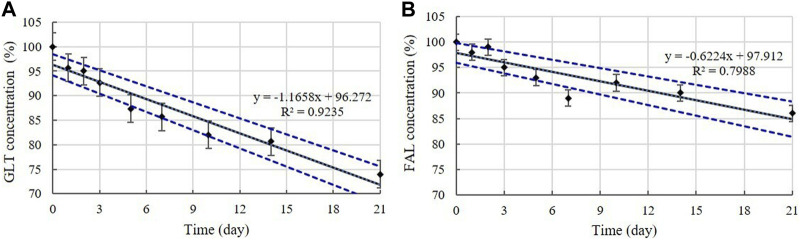
Linear regression with 95% confidence intervals for GLT and FAL at 45°C. **(A)** GLT concentration. **(B)** FAL concentration. Concentration is expressed as a percentage of the concentration measured at study initiation. Linear trend line fit (solid line), and the lower and upper 95% CI (dotted line) of the data are displayed.

Disinfectants C and F are QAC-based disinfectants combined with the aldehydes GLT and FAL. GLT and FAL were present in the diluted disinfectant solutions of disinfectant C at concentrations of 0.033% and 0.053%, respectively. GLT was present in the diluted disinfectant solutions of disinfectant F at a concentration of 0.234% (Table 1). Disinfectant R contained GLT as the single active ingredient, which had a concentration of 0.200% in the diluted disinfectant R solution (Table 1). The concentrations in the samples did not change significantly at 4, 20, and 30°C during the experimental storage period. However, the contents of both GLT and FAL decreased significantly (*p* < 0.05) at 45°C during the experimental storage period. GLT exhibited a 51, 10, and 17% content reduction in the diluted disinfectants C, F, and R solutions after 21 days (Figure 6), whereas FAL exhibited a 14% content reduction in the diluted disinfectant C solution (Figure 6). Furthermore, the lower level of the 95% CI was below 90% of the initial concentrations on Days 3 and 8 for GLT and FAL at 45°C, respectively (Figure 7).

##### 3.2.2.2 PPMS content

**FIGURE 8:**
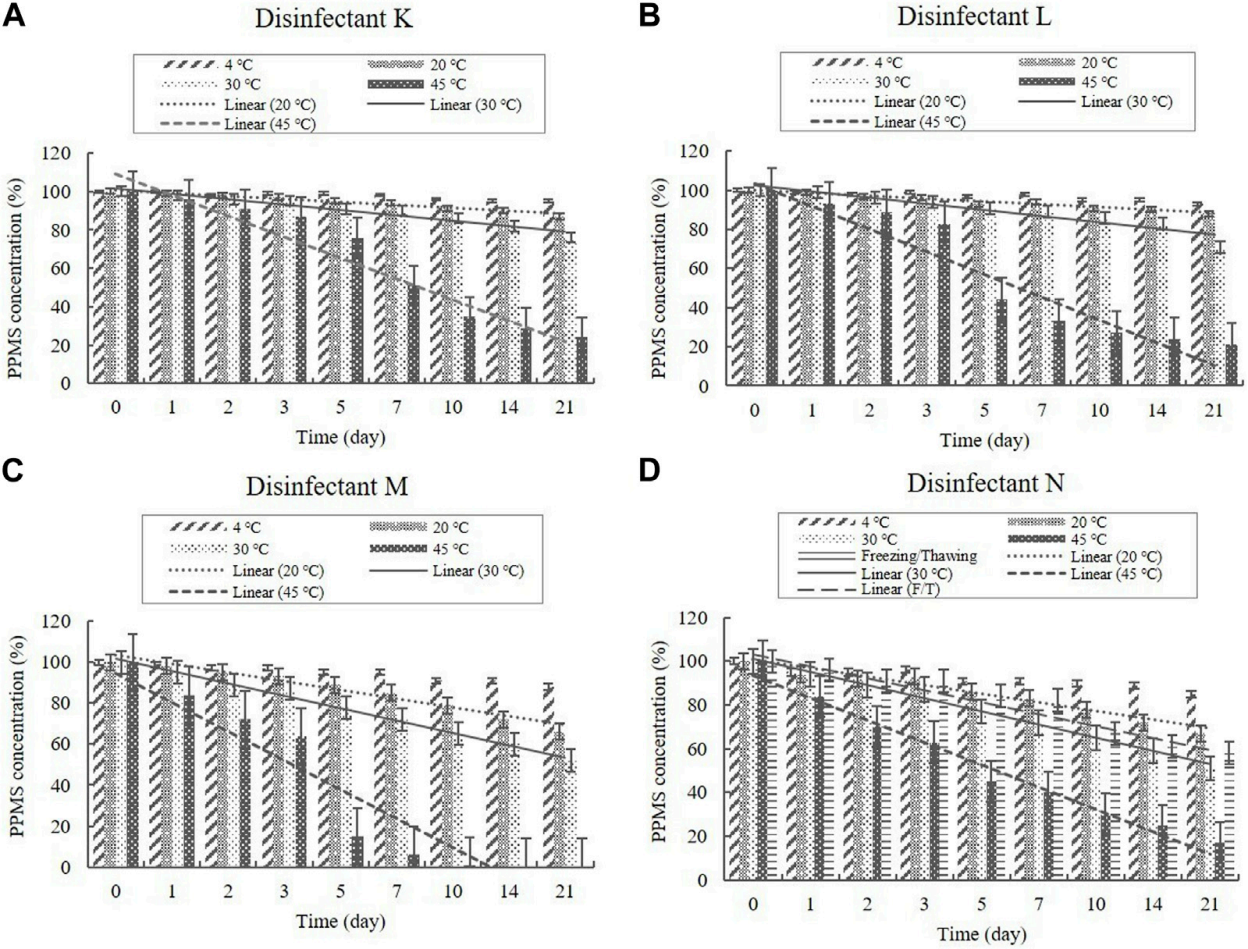
Stability of potassium peroxymonosulfate (PPMS) at various temperatures over time. PPMS concentration in the diluted **(A)** disinfectant K solution, **(B)** disinfectant L solution, **(C)** disinfectant M solution, and **(D)** disinfectant N solution. Concentration is expressed as a percentage of the concentration measured at study initiation. Linear trendline fit of each temperature data is displayed.

**FIGURE 9:**
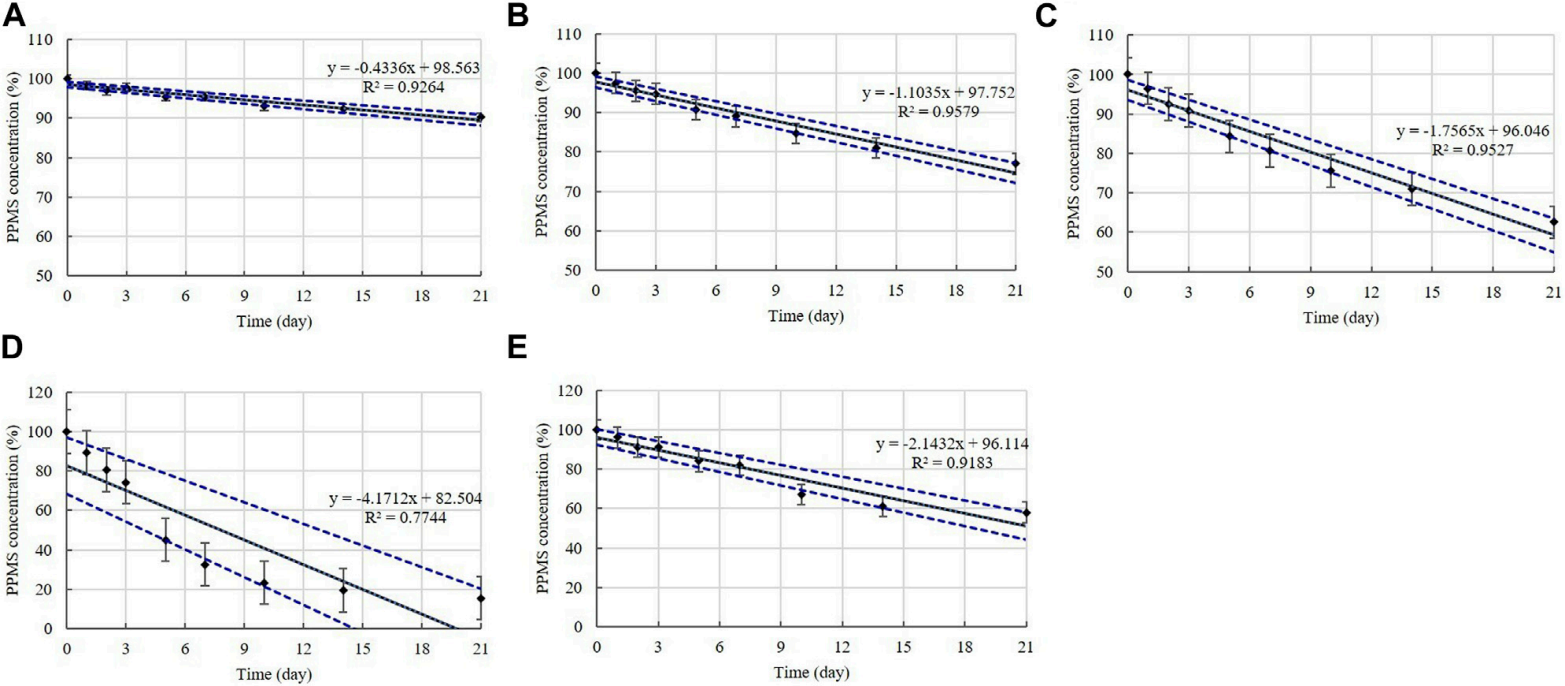
Linear regression with 95% confidence intervals for PPMS. PPMS concentration at: **(A)** 4°C, **(B)** 20°C, **(C)** 30°C, **(D)** 45°C, and **(E)** under freezing/thawing cycle. Concentration is expressed as a percentage of the concentration measured at study initiation. Linear trendline fit (solid line), and the lower and upper 95% CI (dotted line) of the data are displayed.

Disinfectants K, L, M, and N are PPMS-based disinfectants with an active ingredient either alone or combined with an acid. PPMS was present in the diluted disinfectant solutions at concentrations of 0.250%–0.500% (Table 1). Samples taken from the diluted disinfectant solutions exhibited a drastic decrease in the PPMS concentration in a time-temperature-dependent manner, i.e., the concentration decreased over time as the temperature increased. After 21 days, the PPMS concentration measured, on average, 90.25% of its initial concentration at 4°C; however, at 20, 30, and 45°C, the concentration measured, on average, lower than 90% of its initial concentration on Days 7, 5, and 1, respectively. When exposed to a freezing/thawing cycle, the PPMS concentration in the diluted disinfectant N solution measured lower than 90% of its initial concentration on Day 5 (Figure 8). The lower 95% CI displayed below 90% of the initial concentrations on Day 5 at 20°C, on Day 2 at 30°C, and on Day 1 under a freezing/thawing cycle. The degradation of PPMS at 45°C occurred too quickly to enable an accurate stability assessment (Figure 9).

##### 3.2.2.3 MA content

**FIGURE 10:**
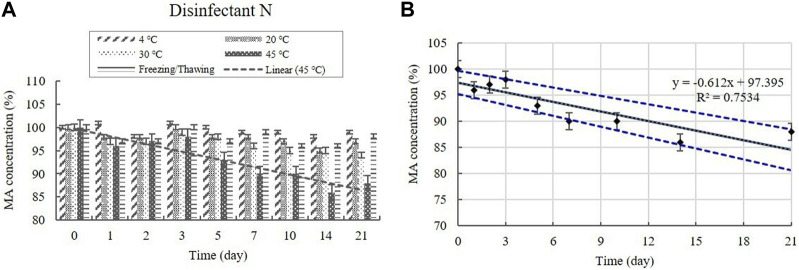
**(A)** Stability of malic acid (MA) at various temperatures over time. MA concentration in the diluted disinfectant N solution. Linear trendline fit of each temperature data are displayed. **(B)** Linear regression with 95% confidence intervals for MA at 45°C. Concentration is expressed as a percentage of the concentration measured at study initiation. Linear trendline fit (solid line), and the lower and upper 95% CI (dotted line) of the data are displayed.

The MA content in the diluted disinfectant N solution was measured using HPLC at a concentration of 0.100% (Table 1). The changes in the MA concentration of the samples did not significantly differ at 4, 20, and 30°C during the experimental storage period, nor under the freezing/thawing cycle (Figure 10). However, the concentration decreased significantly (*p* < 0.05) at 45°C during the experimental storage period, showing a 12% reduction in MA concentration**.** The lower 95% CI displayed below 90% of the initial concentration on Day 7 at 45°C (Figure 10).

##### 3.2.2.4 PAA content

**FIGURE 11:**
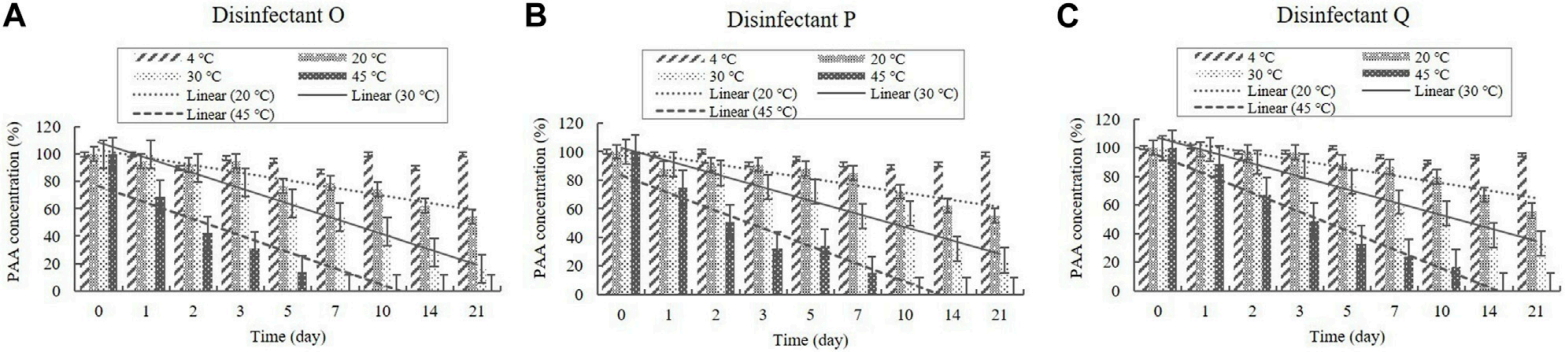
Stability of peracetic acid (PAA) at various temperatures over time. PAA concentration in the diluted **(A)** disinfectant O solution, **(B)** disinfectant *p* solution, and **(C)** disinfectant Q solution. Concentration is expressed as a percentage of the concentration measured at study initiation. Linear trendline fit of each temperature data is displayed.

**FIGURE 12:**
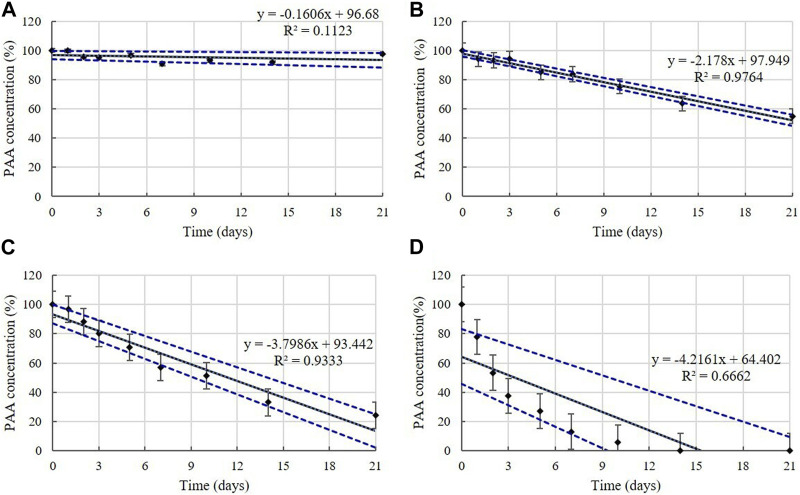
Linear regression with 95% confidence intervals for PAA. PAA concentration at: **(A)** 4°C, **(B)** 20°C, **(C)** 30°C, and **(D)** 45°C. Concentration is expressed as a percentage of the concentration measured at study initiation. Linear trendline fit (solid line), and the lower and upper 95% CI (dotted line) of the data are displayed.

PAA is present in the diluted solutions of disinfectants O, P, and Q at concentrations of 0.006%–0.010% (Table 1). The concentrations in the samples were >90% of the initial concentrations at 4°C during the experimental storage period (Figure 11). However, samples taken from the diluted disinfectant solutions exhibited a drastic decrease in PAA concentrations over time as the temperature increased. After 21 days, the PAA concentration measured, on average, 55% of its initial concentration at 20°C, whereas at 30°C and 45°C it measured, on average, below 90% of its initial concentration on Days 2 and 1, respectively (Figure 11). The lower 95% CI indicates that the degradation of PAA at 20, 30, and 45°C occurred too quickly enable an accurate stability assessment (Figure 12).

## 5 Discussion

Effective disinfection is dependent on the correct disinfectant concentration. Using a concentration lower than the recommended level may affect the efficacy of the disinfectant. Conversely, over-concentrations result in disinfectant waste, which can cause serious harm to people, livestock, and the environment. Disinfectants are typically dispersed in the environment after dilution, and temperature is critical in determining the duration of their effectiveness. Typically, the effectiveness of the disinfectant decreases at temperatures below 15°C and above 20°C (Kim et al., 2013). The chemical potency of disinfectants decreases at low temperatures (Lin et al., 2020), making disinfection in cold climates a huge challenge. To compensate for any loss of efficacy under colder conditions, the disinfectant dosage is usually increased. Kim et al. (2020) conducted an on-site assessment of livestock disinfection facilities from winter to early spring (February to May 2017) in South Korea, and reported that 93.9% of all samples of the diluted disinfectant solutions, used over several days, had either inadequate concentrations or over-concentrations of active ingredients. In some cases, no active ingredients were detected in the solutions.

The stability of reactive species is critical in determining the inactivation effectiveness and shelf life of diluted disinfectant solutions. The chemical stability and disinfection potential of disinfectants are affected by storage conditions; however, the influence of such storage conditions on active ingredients is unclear and little information has been published on the stability of diluted disinfectant solutions under simulated storage conditions.

The stability of a chemical product is affected by the physical changes it undergoes due to temperature fluctuations, such as freezing and thawing (Bajaj et al., 2012). The potential adverse effects of the instability of chemical products, based on content changes of the active ingredients, are: 1) degradation of the active ingredient to less than 90% of the label claim (unacceptable quality), 2) increase in concentration of the active ingredient due to solvent escape or evaporation, caused by insufficiently sealed packaging (container), and 3) loss of content uniformity due to loss of content as a function of time (Bajaj et al., 2012). Some disinfectants lose stability quickly upon preparation for use, whereas others are unstable over long storage periods, particularly in the presence of heat (Dvorak, 2008).

Stability testing is used to determine how the quality of chemical ingredients fluctuates over time when exposed to a range of environmental conditions, so that storage conditions can be prescribed and the shelf life of chemicals can be established (Farrag et al., 2023). In this study, we performed concentration-based assays to test the stability of the active ingredients in diluted disinfectant solutions under varying storage conditions. The data indicated that the active ingredients, such as QACs, CA, AA, PA, H_2_O_2_, NaOCl, and CuSO_4_.5H_2_O, retained 90% of their initial concentration for the 21-day study period at storage temperatures of 4, 20, 30, and 45°C. In addition, QACs and CA were stable during freezing/thawing cycles. QACs consist of only stable chemical bonds, such as C–N, C–C, and C–H, which make their natural degradation rather difficult (Zhang et al., 2021). A recently published study presented the thermal stability of an aqueous solution of QACs up to 200°C (Zhuravlev et al., 2022). Tembo et al. (2017) reported that the CA content in juice samples was generally not strongly influenced by storage temperatures (freezing, 4, 6, 15, and 30°C) for 60 days, and that the decrease in CA content was significantly lower (*p* ≤ 0.05) under refrigeration storage (6°C) than at elevated temperatures (15°C and 30°C). The effects of temperature on the decomposition of disinfectant solutions vary; however, the concentration of active species in AA and PA solutions typically decreases, and their critical temperatures are 230°C and 450°C, respectively (Li et al., 2017; Joseph and White, 2021; Xu et al., 2021). H_2_O_2_, one of the most accessible disinfectants in various applications, has a limited stability, which markedly decreases the expiration period of a disinfectant (Madanská et al., 2004). Storage under decreased temperature proved to be more suitable in terms of content stability of H_2_O_2_ (Madanská et al., 2004), which is consistent with our result that showed that a storage temperature of ≤ 30°C is favorable for a 21-day stability. Gélinas et al. (1984) reported that GLT is much more affected by temperature than NaOCl, thus confirming our results, and that NaOCl is a better disinfectant at low temperatures. Chlorine compounds lose potency over time and are not active at temperatures above 110°F (43.3°C) (Dvorak, 2008). In our study, only one sample was investigated, and no fluctuation in concentrations was observed at ≤ 45°C storage temperatures for the study period. However, chlorine compounds are rapidly inactivated by light and certain metals; thus, fresh solutions should always be used (Dvorak, 2008). According to Cheng et al. (2019), the thermal decomposition reaction of CuSO_4_.5H_2_O is initiated from 50°C, which is consistent with our results of stable conditions at ≤ 45°C storage temperatures.

The aldehydes, GLT and FAL, and MA retained 90% of their initial concentrations at 4, 20, and 30°C for 21 days; however, degradation patterns were observed when stored at 45°C, thus suggesting that they are unstable at high temperatures. In particular, MA was affected more by high-temperature changes than by freezing/thawing cycles. A previous study reported that MA content in juice samples was generally not strongly influenced by storage temperature (freezing, 4, 6, 15, and 30°C) for 60 days (Tembo et al., 2017) and that the decrease in MA was significantly lower (*p* ≤ 0.05) under refrigerated storage (6°C) than at elevated temperatures (15°C and 30°C). GLT is an active ingredient affected by temperature (Gélinas et al., 1984; Russell, 2008). Previous data suggest that the concentration of GLT in hospital biocides appeared to be reduced to 50% after incubation for 2 w at 40°C, whereas smaller variations were observed in the sample incubated at room temperature (Matei et al., 2020). Kim et al. (2013) also reported that the effect of the GLT agents is reduced at temperatures above 20°C. The volatile organic compound FAL is used directly in aqueous solutions as an active ingredient in disinfectants. Increases in temperature and humidity accelerate the release of FAL into the atmosphere because the temperature increases the kinetic energy and accelerates the emission rate of FAL molecules (Liu et al., 2017; Zhang et al., 2022). FAL decomposes into methanol and carbon monoxide at temperatures above 150°C, and the ideal temperature for storage of FAL solutions is between 15°C and 25°C (WHO, 1991). Liu et al. (2017) found that the FAL ventilation increased 10%–30% when the temperature was increased by 5°C. Zhang et al. (2022) found that the FAL ventilation at 60°C was more than ten times of that at 20°C. The FAL content of our sample stored at high temperatures indicated that the FAL in the sample was released into the air as a gas upon heating the solution. Considering the results obtained in this and other studies, diluted disinfectant solution that contain GLT, FAL, and MA as active ingredients should be stored at ≤ 30°C for chemical stability.

PPMS and PAA, are used as oxidizing agents. In this study, the concentration of PAA and PPMS decreased over time with increasing temperature. A stability of 90% was shown at 4°C but not under other conditions. Kim et al. (2013) reported that, at 15°C–20°C, oxidizing agents are active but chemically unstable in an aqueous solution. To maintain stability, PAA should preferably be stored at low temperatures in its original containers (Block, 2001). Diluted PAA solutions (1%) lose 50% of their sanitation power within 6 days (Kunigk et al., 2001). Increasing the temperature causes a more rapid decomposition. For example, the concentration decreased by 50% in 72 h at 45°C, whereas the loss was only 33% in 240 h at 25°C (Kunigk et al., 2001), indicating that temperature plays an important role in the shelf life of PAA solutions. Li et al. (2021) reported that the composition temperature of PPMS is low, and its activity is not stable over long periods in aqueous solutions. PPMS is relatively stable as a salt at ambient temperatures (Suresh et al., 2019); however, Sykes and Weese (2014) demonstrated that a solution prepared from powder remained active for only 7 days. The decomposition of PPMS is exothermic and can accelerate if conditions allow the product temperature to rise; therefore, packaging temperature should not exceed 30°C (Gant and Barr, 2006). The results of this study and those of previous studies demonstrate that disinfectants containing a combination of H_2_O_2_ and PAA as well as PPMS disinfectants should be used within a short period after dilution. In addition, heating as a means to prevent freezing of the disinfectants in winter should be avoided, as heat reduces the PAA and PPMS contents.

The reactive species have to remain stable during storage to maintain their disinfection properties (Tsoukou et al., 2020). If the concentration of the active ingredient in the disinfectant decreases, the contact time may be increased to achieve effective disinfection. However, this may not always hold true. Thus, when selecting the best disinfectant and optimal conditions for maximum efficacy, the temperature of use and storage conditions should be considered, as well as the active ingredient concentration in the solution. Herein, the results presented provide a basis for the storage of diluted veterinary disinfectant solutions and practical applications for disinfection in the veterinary field. Despite the current lack of data and relative complexity of this subject, striving towards a better understanding of the effects of temperatures and storage periods on diluted disinfectant solutions will ultimately lead to an improved field guidance for the selection and use of a seasonally appropriate veterinary disinfectants and their storage conditions. To the best of our knowledge, our study is the first to have investigated the chemical stability of active ingredients in diluted veterinary disinfectants under simulated storage conditions.

## Data Availability

The original contributions presented in the study are included in the article/Supplementary Material, further inquiries can be directed to the corresponding author.
