# Supplementary material for: Chemical stability of active ingredients in diluted veterinary disinfectant solutions under simulated storage conditions
Source: Front Chem. 2023 Jun 16;11:1204477. doi: 10.3389/fchem.2023.1204477 (PMC10311561; doi:10.3389/fchem.2023.1204477)
Supplement: Supplementary file 1 [file DataSheet1.docx]

Supplementary Material

Chemical stability of active ingredients in diluted veterinary disinfectant solutions under simulated storage conditions

Chae Hong Rhee, Hye-sook Lee, Hyeong-jun Yun, Ga-Hee Lee, Su-Jeong Kim, Sok Song, Myoung-Heon Lee, Moon Her, Wooseog Jeong*

*** Correspondence:** Wooseog Jeong: wjeong@korea.kr

# Supplementary Figures and Tables

## Supplementary Figures


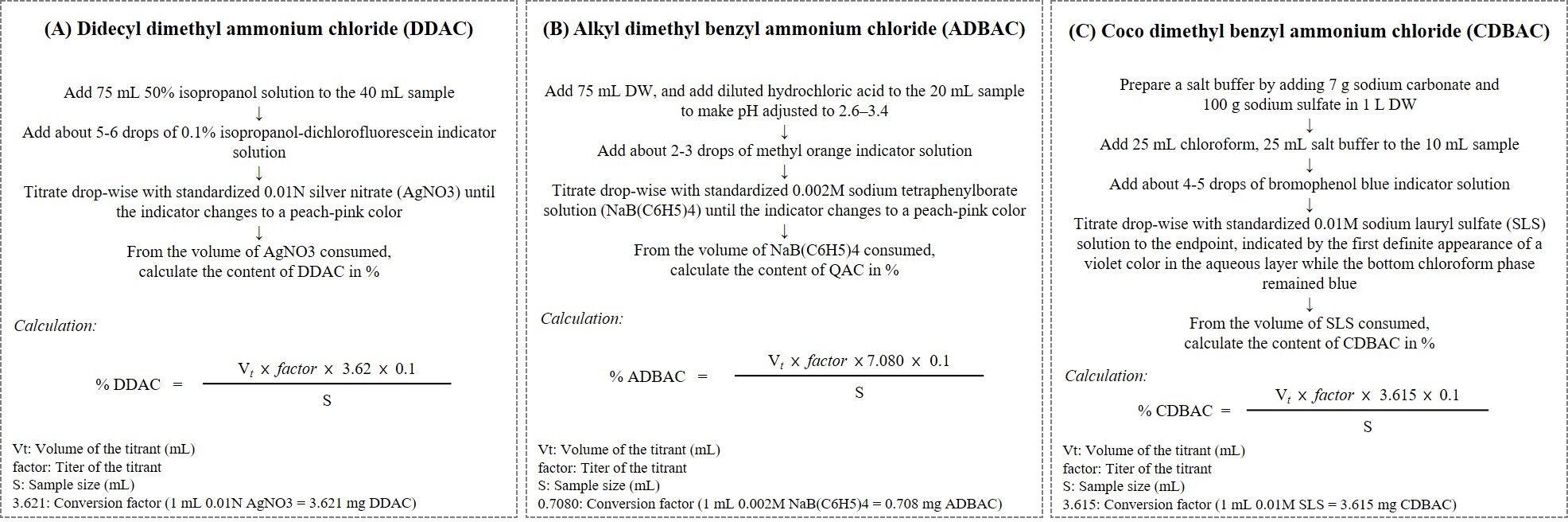


**Supplementary Figure S1.** Flowchart showing the procedure of the titration method for determination of quaternary ammonium compounds: (A) didecyl demethyl ammonium chloride (DDAC), (B) dimethyl benzyl ammonium chloride (ADBAC), and (C) coco dimethyl benzyl ammonium chloride (CDBAC).


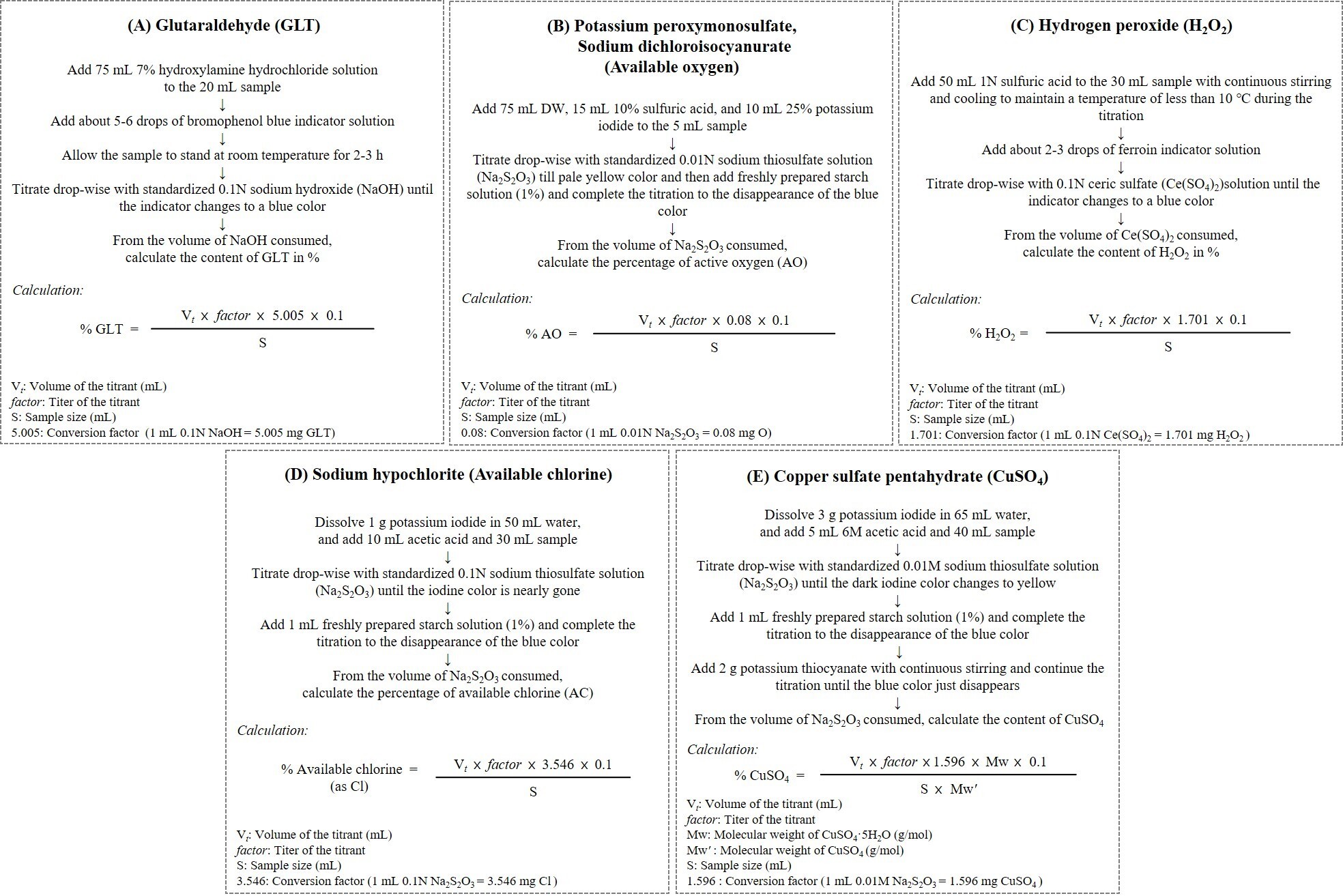


**Supplementary Figure S2.** Flowchart showing the procedure of the titration method for determination of (A) glutaraldehyde (GLT), (B) active oxygen (AO) for potassium peroxymonosulfate and sodium dichloroisocyanurate, (C) hydrogen peroxide (H_2_O_2_), (D) active chloride (AC) for sodium hypochlorite, and (E) copper sulfate (CuSO_4_) for copper sulfate pentahydrate (CuSO_4_·5H_2_O).
